# TiLIA: a software package for image analysis of firefly flash patterns

**DOI:** 10.1002/ece3.2078

**Published:** 2016-03-30

**Authors:** Junsuke Konno, Yoko Hatta‐Ohashi, Ryutaro Akiyoshi, Anchana Thancharoen, Somyot Silalom, Watana Sakchoowong, Vor Yiu, Nobuyoshi Ohba, Hirobumi Suzuki

**Affiliations:** ^1^ Advanced Analysis Technology DepartmentOlympus CorporationKuboyama 2‐3HachiojiTokyo192‐8512Japan; ^2^ Department of EntomologyFaculty of AgricultureKasetsart UniversityChatuchakBangkok10900Thailand; ^3^ Entomology SectionQueen Sirikit Botanic GardenMaerimChiang Mai50180Thailand; ^4^ Department of National Parks, Wildlife and Plant ConservationForest and Plant Conservation Research OfficeChatuchakBangkok10900Thailand; ^5^ Hong Kong Entomological SocietyYuen Long, Hong KongChina; ^6^ The Ohba Firefly InstituteMabori‐kaigan 4‐1‐12‐204YokosukaKanagawa239‐0801Japan

**Keywords:** Flash communication, flight path tracing, time‐lapse image analysis

## Abstract

As flash signaling patterns of fireflies are species specific, signal‐pattern analysis is important for understanding this system of communication. Here, we present time‐lapse image analysis (TiLIA), a free open‐source software package for signal and flight pattern analyses of fireflies that uses video‐recorded image data. TiLIA enables flight path tracing of individual fireflies and provides frame‐by‐frame coordinates and light intensity data. As an example of TiLIA capabilities, we demonstrate flash pattern analysis of the fireflies *Luciola cruciata* and *L. lateralis* during courtship behavior.

## Introduction

Light production in adult fireflies is a mating adaptation (McDermott 1910; Mast 1912; Barber and McDermott 1951), and species‐specific mating‐related flash signals play a role in reproductive isolation (Lloyd 1966). Thus, signal‐pattern analysis is important for understanding flash communication systems of the firefly.

In the earliest studies, the relative intensities and pulse duration of firefly flash signals and behaviors were estimated and observed by eye (Osten‐Sacken 1861; Emery 1887; McDermott 1910; Mast 1912), and McDermott (1914, 1917) drew a diagram of male and female flash patterns of some firefly species. Since the middle 1900s, several methods for recording firefly flash patterns have been used. Barber and McDermott (1951) measured the pulse duration and frequency of flashes by stopwatch and subjectively described the intensity and color of the flashes. Lloyd (1966, 1969) developed a recording system in which the flash was detected by a photo‐sensor, and the light intensity was transduced to a frequency‐modulated audio signal, which was recorded on a magnetic tape. The recorded tone was then transduced to direct current voltage that was fed into an oscilloscope and a chart recorder. With this system, which was used for observations in both the laboratory and the field (Seliger et al. 1964; Buck and Buck 1968; Papi 1969; Lloyd 1973, 1984; Ohba 1979, 1983), the female response to male or artificial signals could be analyzed within 10 msec. However, the working distance of the photo‐sensor and the ability to analyze multiple individuals simultaneously were limited.

The recording of flash images on film was attempted (Buck and Buck 1968; Ohba 1980), but insufficient photosensitivity of the film restricted this approach to the analysis of flash intervals only. Since that time, video recording coupled with an image intensifier has allowed flash signals to be analyzed in more detail (Ohba 1984). The flight path of the firefly can be traced with a hand‐held photo‐sensor manually on a monitor screen, and the light intensity of a firefly can be transduced to direct current voltage that is fed into a chart recorder or a personal computer (Makino et al. 1994; Copeland and Moiseff 1995). With this system, multiple studies have described flash patterns of numerous firefly species together with their behavior and ecology (Copeland and Moiseff 1995, 1997; Ohba et al. 1995, 2001; Moiseff and Copeland 2000; Ohba 2000, 2001; Ohba and Wong 2004; Fu et al. 2006; Fu and Ballantyne 2008; Ohba and Shimoyama 2009; Ballantyne et al. 2013). Although flashing behavior is recorded on a videotape, this system was also initially restricted to the analysis of flash intervals.

Recently, the photosensitivity of digital video recorders has improved, and flash patterns of fireflies can be recorded without an image intensifier (Kawano 2009; Yiu 2013). This allows much easier spatiotemporal image analysis of flash patterns by a personal computer. However, commercially available software packages for spatiotemporal analyses of time‐lapse images tend to be too expensive and complicated for use in this simple analysis. Therefore, we developed time‐lapse image analysis (TiLIA), a free open‐source software package for signal and flight pattern analyses of fireflies using video‐recorded image data. TiLIA enables flight path tracing of individual fireflies and provides frame‐by‐frame coordinates and light intensity data chronologically. The method and open‐source application proposed in this study is a step forward into the understanding of flash signal communication in fireflies, species identification, and even for the study of other (marine) organisms using bioluminescent displays for interaction, communication, and/or courtship. As an example of TiLIA capabilities, we performed flash pattern analysis of the fireflies *Luciola cruciata* Motschulsky and *Luciola lateralis* Motschulsky and presented graphical male‐ and female‐specific flash patterns during courtship of *L. lateralis*.

## Materials and Methods

### The TiLIA package

**Figure 1:**
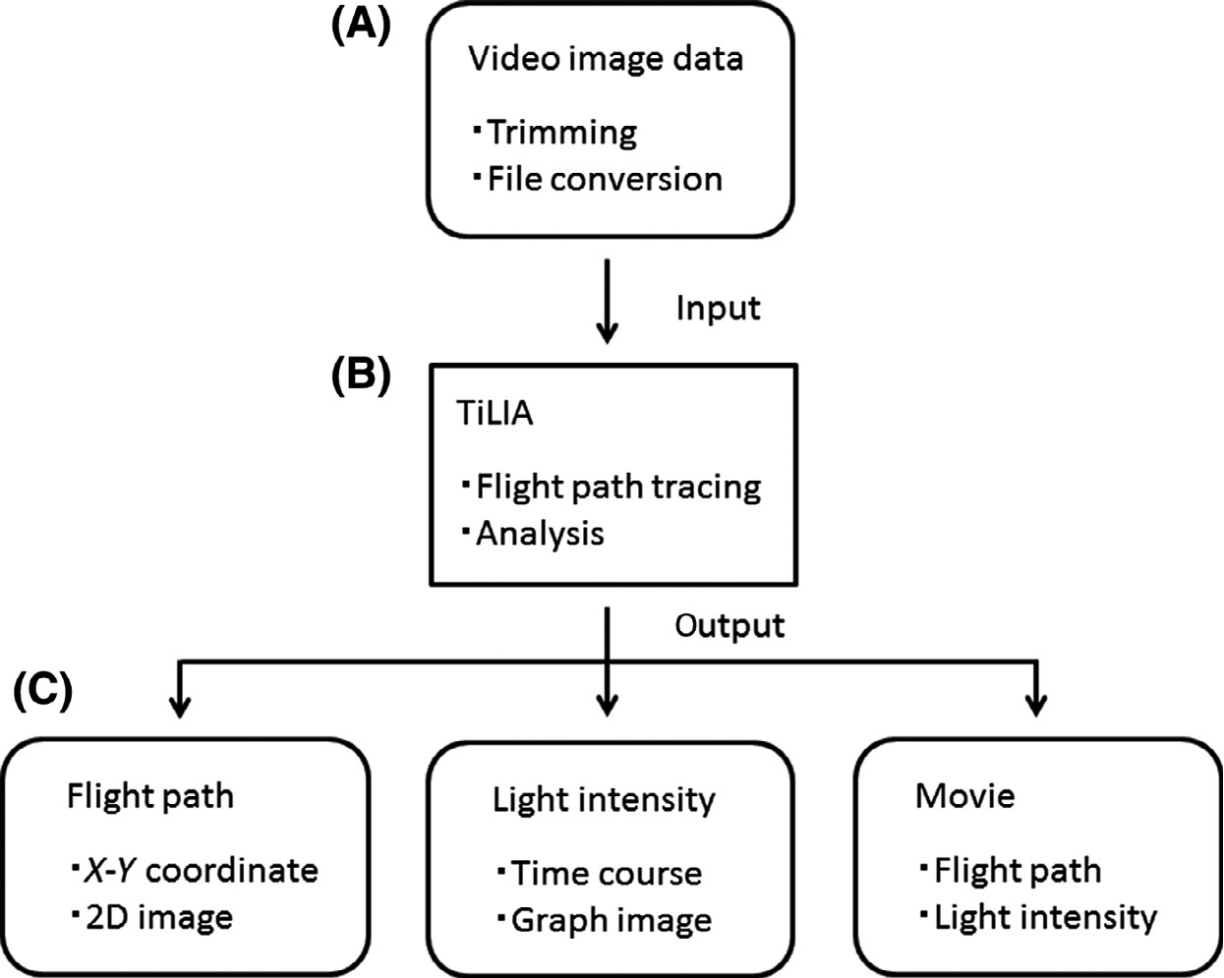
Flowchart for use of TiLIA. (A) A scene of video for analysis is trimmed, and the image file is converted to AVI‐formatted video file. (B) Flight path of the object is traced, and the flight path and light intensity are analyzed. (C) Outputs of flight path, light intensity, and movie are recorded as image, text, and video data.

**Figure 2:**
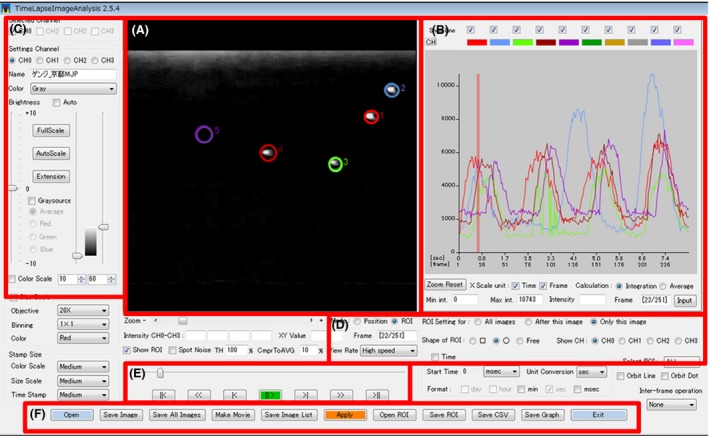
The graphical user interface of TiLIA, which is composed of several spaces and areas: (A) image display space, (B) data analysis space, (C) image property adjustment area, (D) ROI select area, (E) video operation area, and (F) command area. The five ROIs are assigned to *Luciola cruciata* fireflies individually in the image display space (A), and the fireflies are then traced within the ROI manually frame by frame. The time course of light intensity for each ROI is simultaneously displayed as a graph in the data analysis space (B). The flash patterns are described as four flashes of single peak within 8.0 sec except for the blue one (three peaks), and the flash duration and peak interval are 1.3 and 1.9 sec, respectively. The flashes are loosely synchronous. The red vertical line indicates the frame number of the image displayed.

TiLIA is operated with the following three steps (Fig. 1): A) trim a scene of video for analysis and convert it to audio video interleave (AVI)‐format file; B) trace the flight path of object and analyze the flight path and light intensity; and C) record the output results as image, video, and text data. Figure 2 shows the graphical user interface of TiLIA, which is composed of several spaces and areas: A) image display space, B) data analysis space, C) image property adjustment area, D) region of interest (ROI) select area, E) video operation area, and F) command area. An AVI‐format video file of a luminescent object is loaded into TiLIA, and each frame is saved as a separate tagged image file format (TIFF) file. The brightness and contrast of each image are adjusted (original image file is not rewritten), and the appropriate size of the ROI is assigned to an object in the image display space (Fig. 2A). The object is then manually (by mouse) traced frame by frame. This process is very time‐consuming; therefore, a scene selected for analysis is trimmed appropriately using a video editing software or TiLIA in advance. The light intensity value of the ROI in each frame is recorded, and the resulting time course is plotted graphically in the data analysis space (Fig. 2B). The tracing process can be confirmed by viewing the reintegrated video file, and the position of the ROI can be adjusted as necessary. The flight track of the ROI can also be displayed in the video. The graph of time course of light intensity (Fig. 2B) is saved as an image file, and the light intensity values are stored as text data. If flight paths of two fireflies coincide, the two assigned ROIs trace the two paths superpositionally. However, it is impossible to distinguish between the light intensities of two fireflies. Images of the ROI and of flight path, and the ROI coordinates data are also stored.

## Results and Discussion

### Example 1

**Figure 3:**
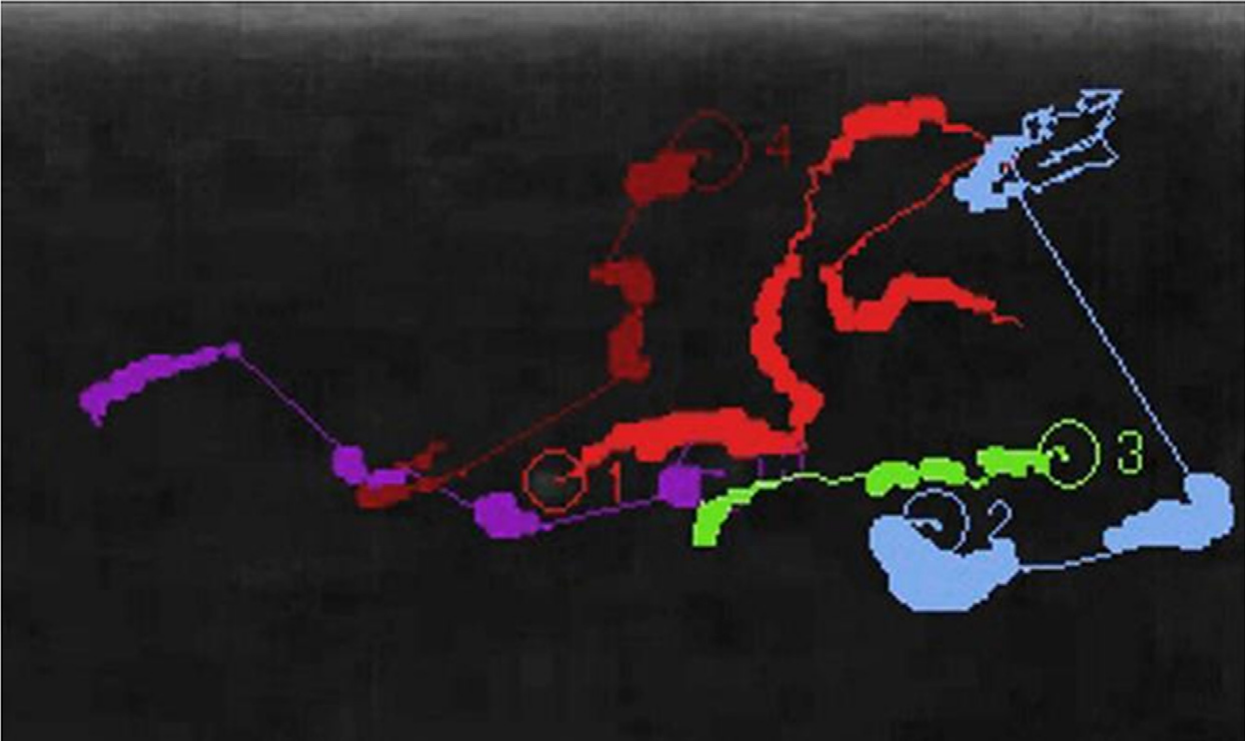
Flight path of male *Luciola cruciata*. Light intensity is indicated by a circle size optimally allocated at seven steps on the orbit line.

*Luciola cruciata* is a species that displays synchronous flashes. After sun set, the males begin to fly, flashing and synchronizing their flashes slowly (Ohba 1983, 1984, 2004). The video of the male's synchronous flashes in the field (Kyoto, Japan) was converted to AVI‐format file and used for analysis by TiLIA. Figure 2A shows an image with assigned ROIs for five males, and Fig. 2B, the graph of time course of light intensity for the five males is indicated by red, brown, green, purple, and blue. The flash patterns were described as four flashes of single peak within 8.0 sec except for the blue one (three peaks). The average flash duration and peak interval were 1.3 and 1.9 sec, respectively. Their flashes were loosely synchronous, as they tended to show flash peaks around the same moment. The flight path was described using an orbit line and the light intensity was indicated with a circle size optimally allocated at seven steps on the orbit line (Fig. 3). With our previous system (Ohba 1984; Makino et al. 1994), it was very difficult to trace multiple individuals with photo‐sensor data because it required rewinding the tape and adjusting the starting point for each individual (i.e., ROI). Image analysis with TiLIA alleviates these issues.

### Example 2

Flash communication and mating behavior of *L. lateralis* have been intensively studied (Ohba 1983, 2004). This communication system can be described in five distinct phases: (1) A flying male emits single‐pulsed flashes and seeks out the female flash pattern that is unique to this species; (2) a female recognizes the male flashes and her interflash interval reduces gradually and the female's unique flash plays an important role in attracting the male; (3) the male approaches the female on the ground and changes his flash pattern to twinkling flashes that, upon visual inspection, appear to be a single flash accompanied with minor blinking flashes; (4) both sexes continue emitting their distinctive flashes, and their interflash intervals are keep on further reducing and the timing of the male–female response flashes does not appear to be critical; and (5) thereafter, they copulate.

**Figure 4:**
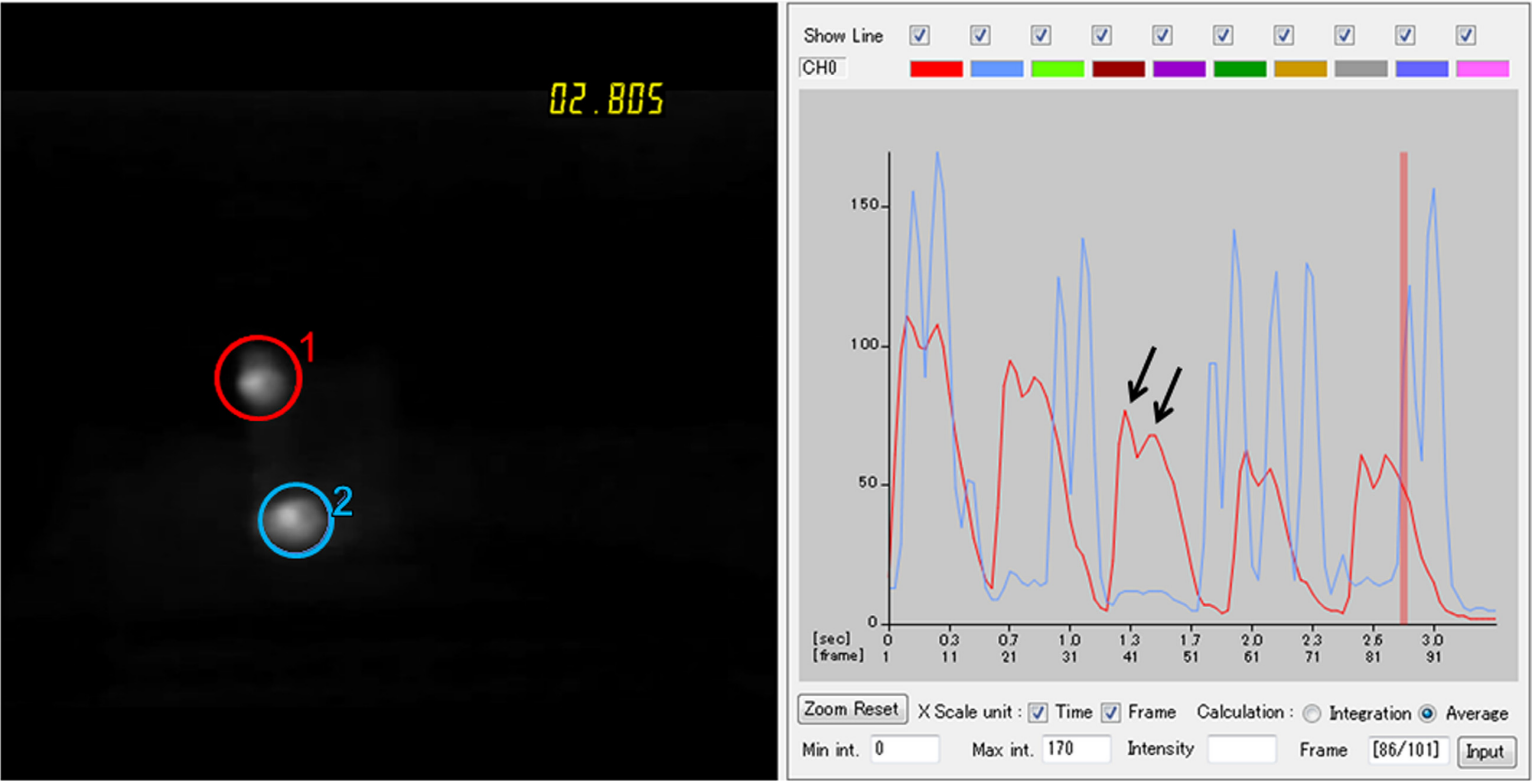
Analysis of flash communication of *Luciola lateralis* by TiLIA. Male and female fireflies are represented by ROIs 1 (red circle) and 2 (blue circle), respectively (left panel), and the time course of light intensities for the two ROIs is displayed as a graph within 3.3 sec (right panel). The male flash pattern (red line) is described as a bimodal double‐pulsed flash (indicated by arrows), and the flash duration, interflash interval, and the bimodal double‐pulsed interval are 566, 624, and 140 msec, respectively. The female flash pattern comprises very rapid double flash, and triple to quadruple flashes. The timer is stamped, sec and msec. The red vertical line indicates the frame number of the image displayed. The integrated video file is provided in Supporting Information, Appendix S1.

A pair of *L. lateralis* was placed in a plastic container, and the flash communication between male and female was recorded by a video camera (Handycam EVCX10, Sony, Tokyo, Japan) equipped with an image intensifier (Star Light Scope, Hamamatsu Photonics, Shizuoka, Japan). The video of the courtship before copulation (phase 4) was converted to an AVI‐formatted file and used for analysis by TiLIA. Figure 4 shows an image with assigned ROIs 1 (red circle) and 2 (blue circle) for male and female fireflies, respectively (left panel), and the graph of the time course of the light intensity of the two ROIs for 3.3 sec (right panel). The male flash pattern (red line) was described as a bimodal double‐pulsed flash (indicated by arrows in the figure). The flash duration, the interflash interval, and the bimodal double‐pulsed interval were 566, 624, and 140 msec, respectively, in average of the five peaks in the graph. The image analysis revealed the twinkling flash recognized by naked eye as a bimodal double‐pulsed flash with an interval of 140 msec. On the other hand, the unique female flash pattern (blue line) was described as a very rapid double flash, triple to quadruple flash. Thus, male‐ and female‐specific flash patterns and the exchange of their flashes during courtship were represented in the graph. The AVI file of male and female activity together with the time‐course analysis is provided in Supporting Information, Appendix S1.

## Further Suggested Uses

TiLIA generates output in the form of time‐series text data of light intensity and coordinates of ROIs. These data can be used for further analyses with other software packages. For example, a power spectrum analysis can be applied to flash pattern data to assess flash–pulse component relationships with behavior or among species (Ohba et al. 1995; Ohba and Shimoyama 2009). A correlation function analysis can be used to assess the degree of synchrony of flashes among individuals. A fractal dimension analysis can be applied to the coordinate data to assess flight pattern characteristics in relation to behavior or environment (Shibue et al. 1995).

## Software Accessibility

The TiLIA software and user's manual can be found at Google Drive (https://drive.google.com/open?id=0B2o7FRVs2VohMmx2QzBVX3ZDeDA).

## Conflict of Interest

None declared.

## Supporting information


**Appendix S1**. The integrated video results from TiLIA that traced male and female *L. lateralis* courtship behavior, with the time‐course analysis of flash patterns.
